# Supplementary material for: Systemic Barriers and Equitable Interventions to Improve Vegetable and Fruit Intake in Children: Interviews with National Food System Actors
Source: Int J Environ Res Public Health. 2019 Apr 17;16(8):1387. doi: 10.3390/ijerph16081387 (PMC6518010; doi:10.3390/ijerph16081387)
Supplement: Supplementary file 1 [file ijerph-16-01387-s001.pdf]

Supplementary Data: Original CMI maps (hand-drawn during interviews)

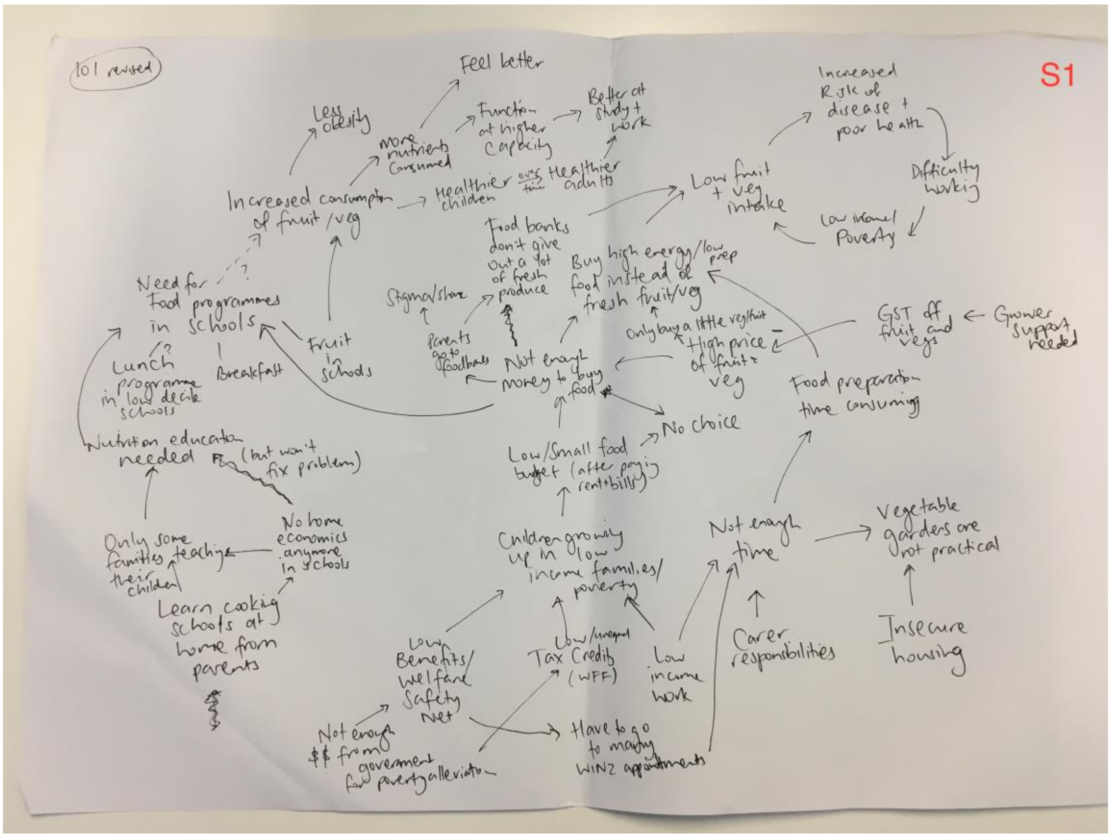


Figure S1. Cognitive map from Interview 101.

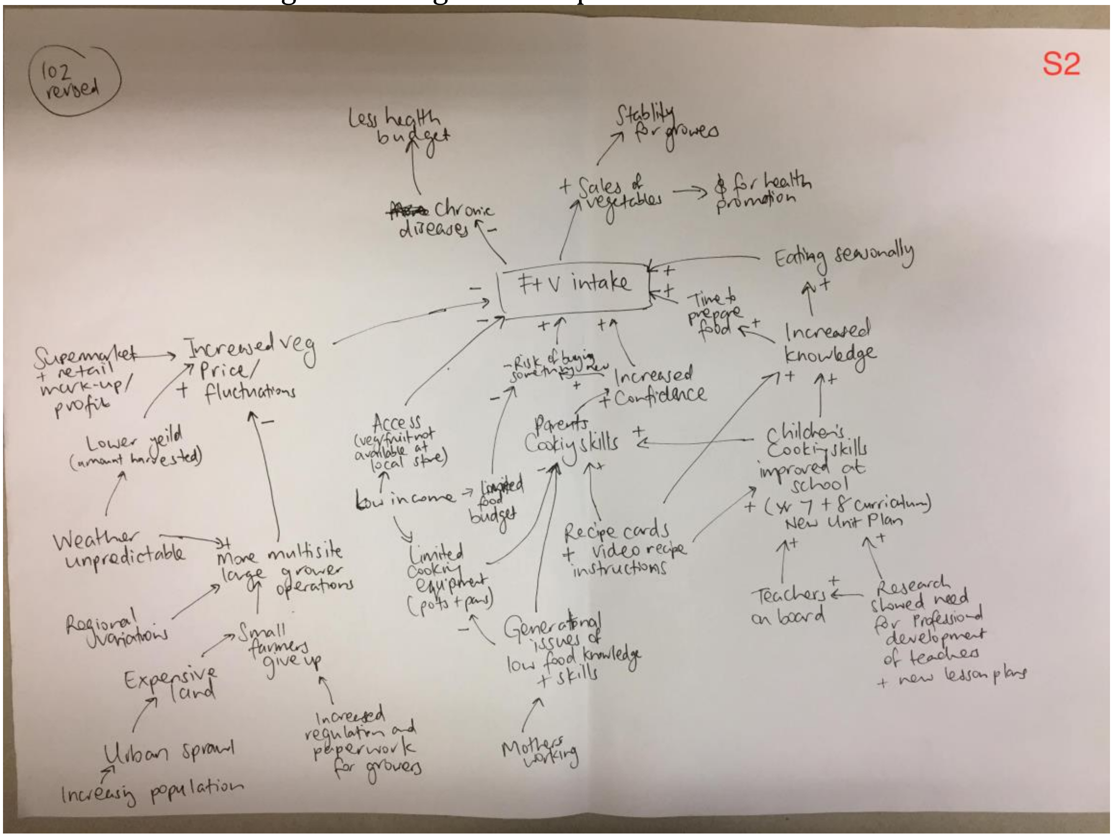


Figure S2. Cognitive map from Interview 102.

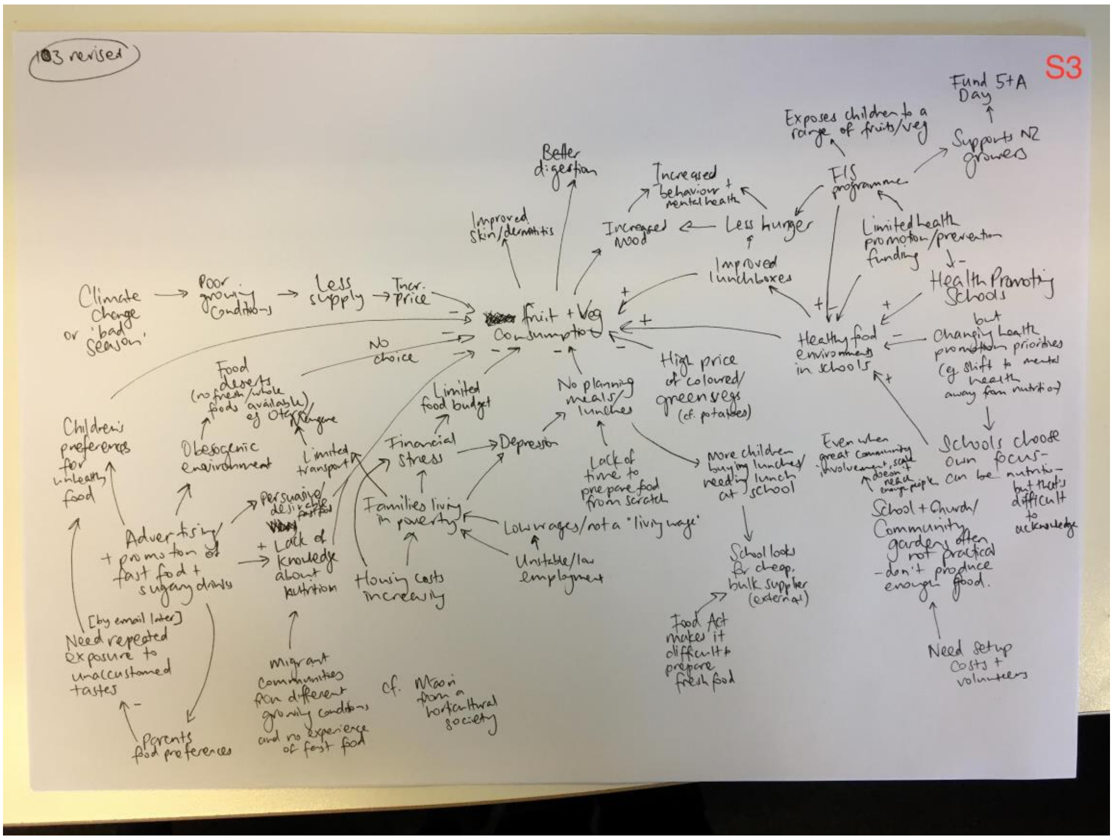


Figure S3. Cognitive map from Interview 103.

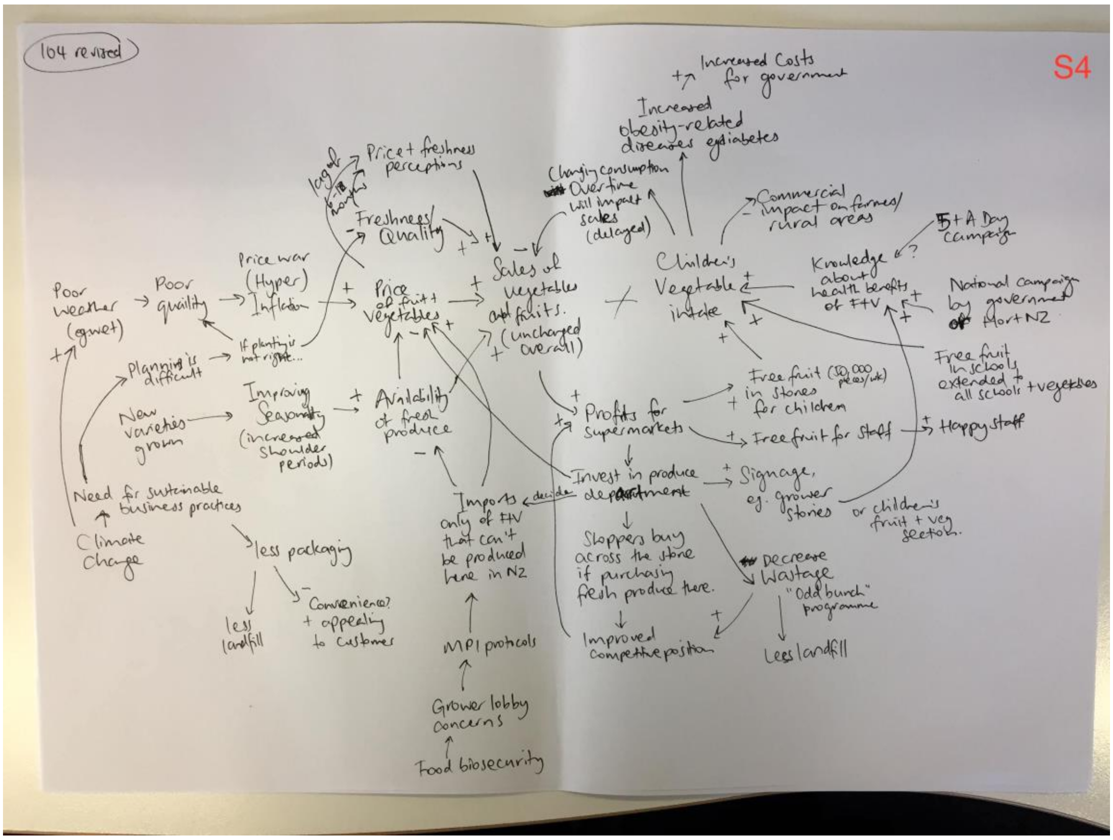


Figure S4: Cognitive map from Interview 104.

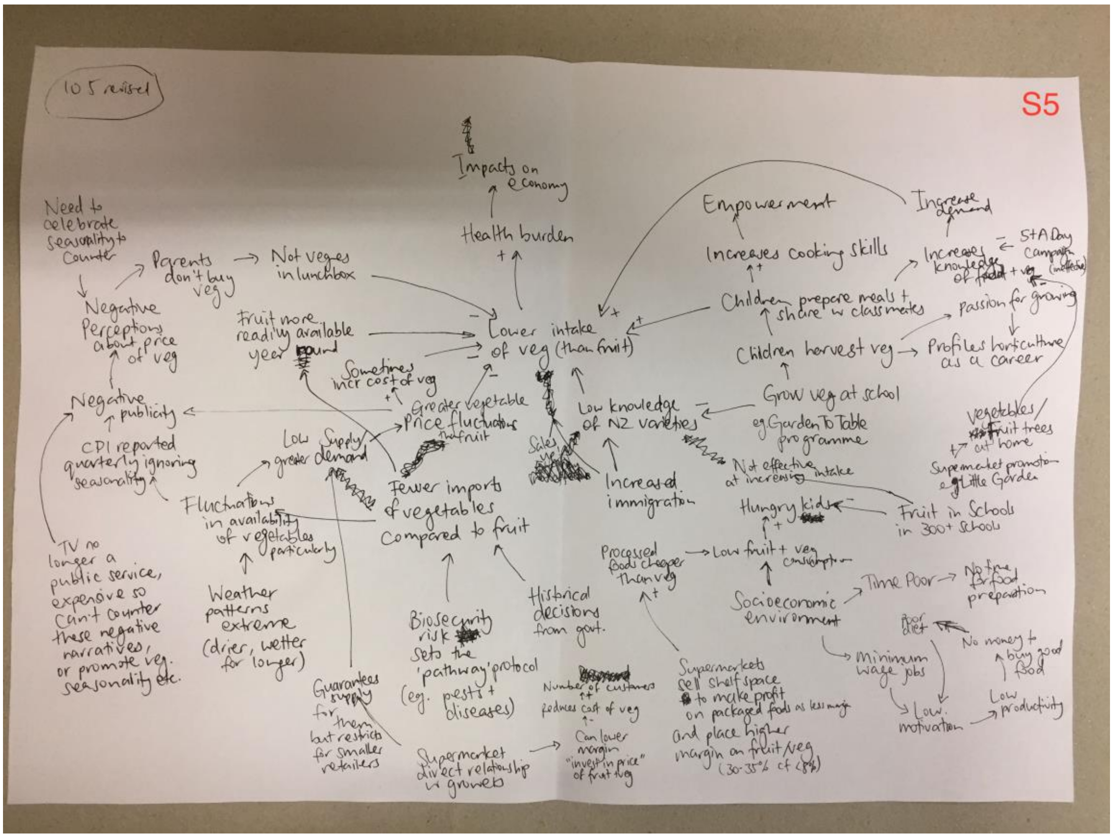


Figure S5. Cognitive map from Interview 105.

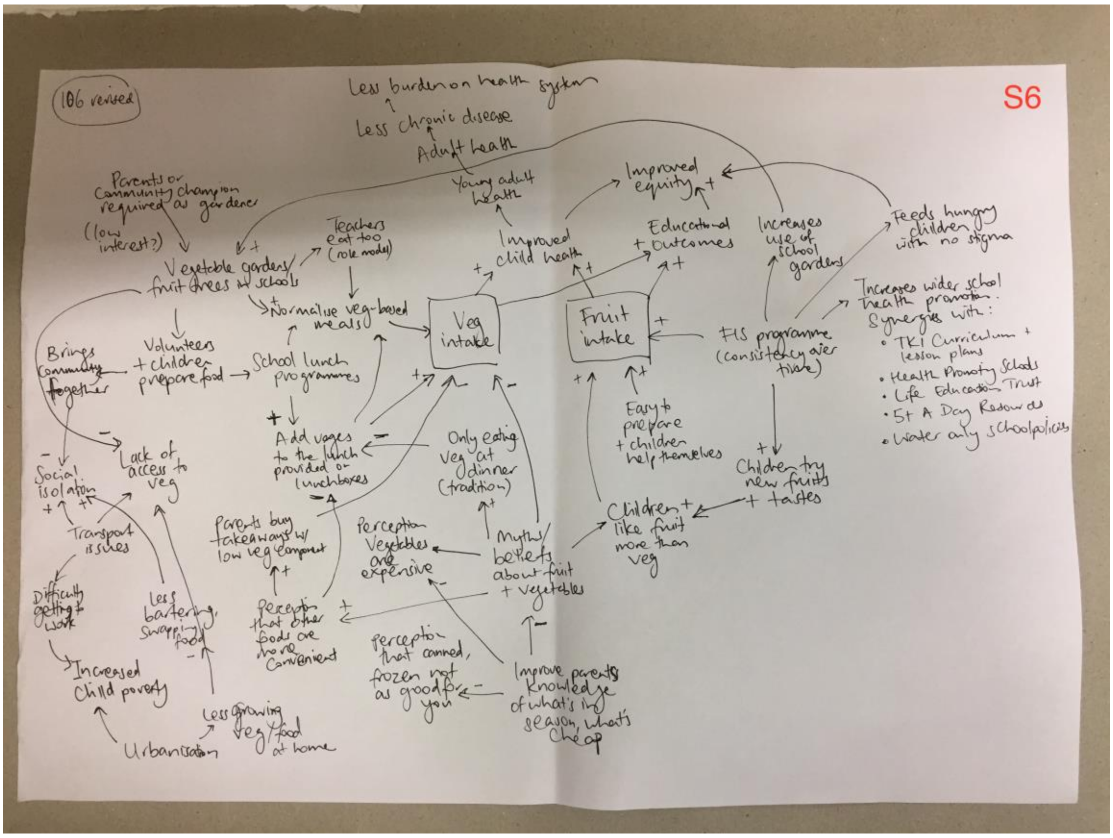


Figure S6. Cognitive map from Interview 106.

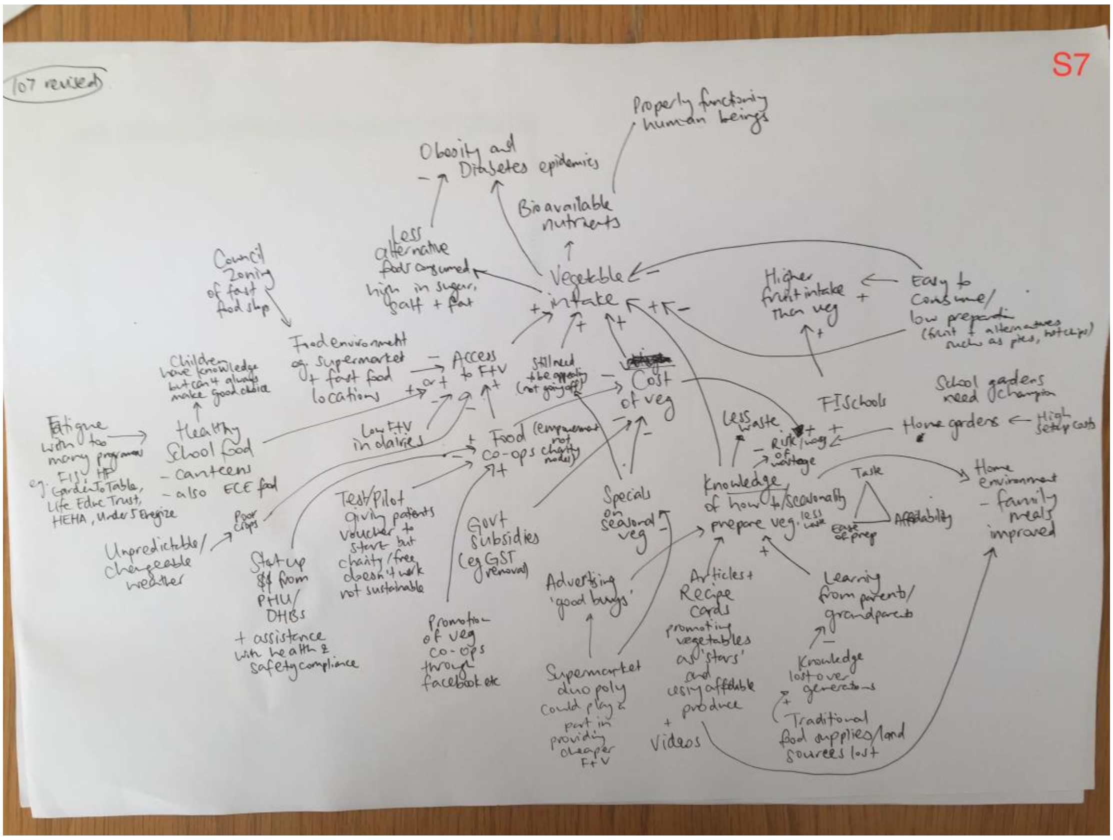


Figure S7. Cognitive map from Interview 107.

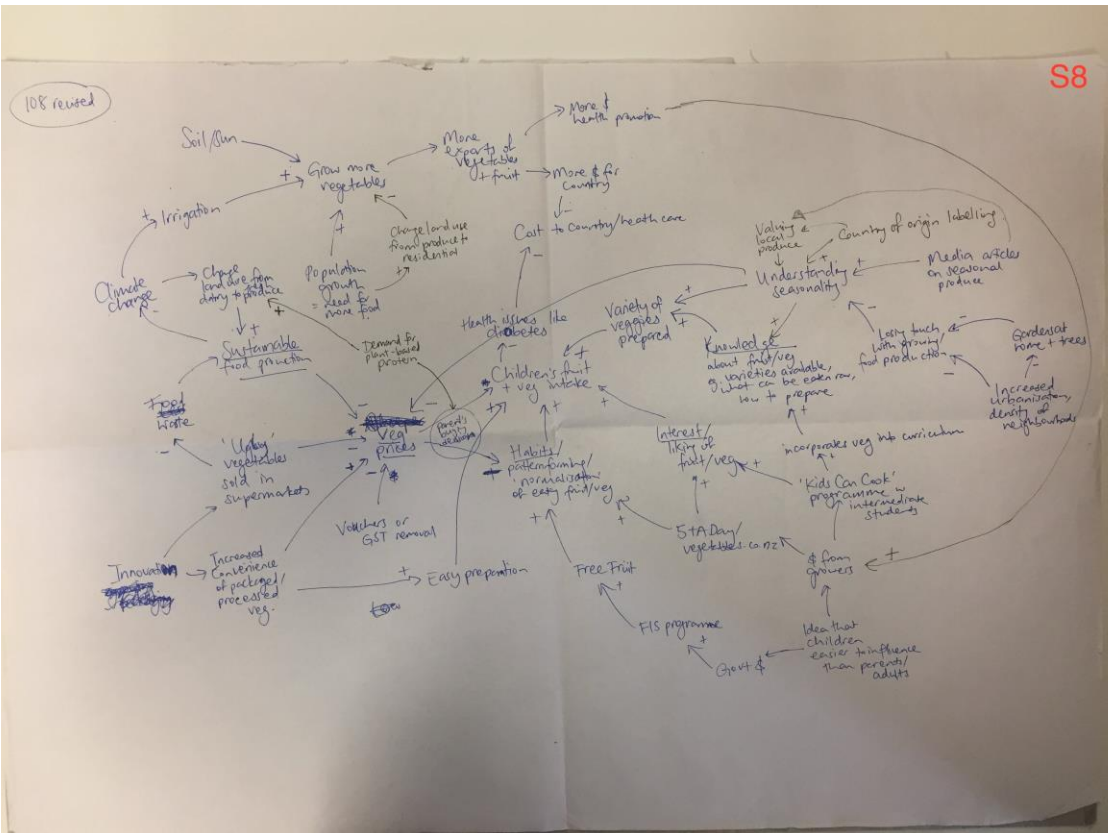


Figure S8. Cognitive map from Interview 108.

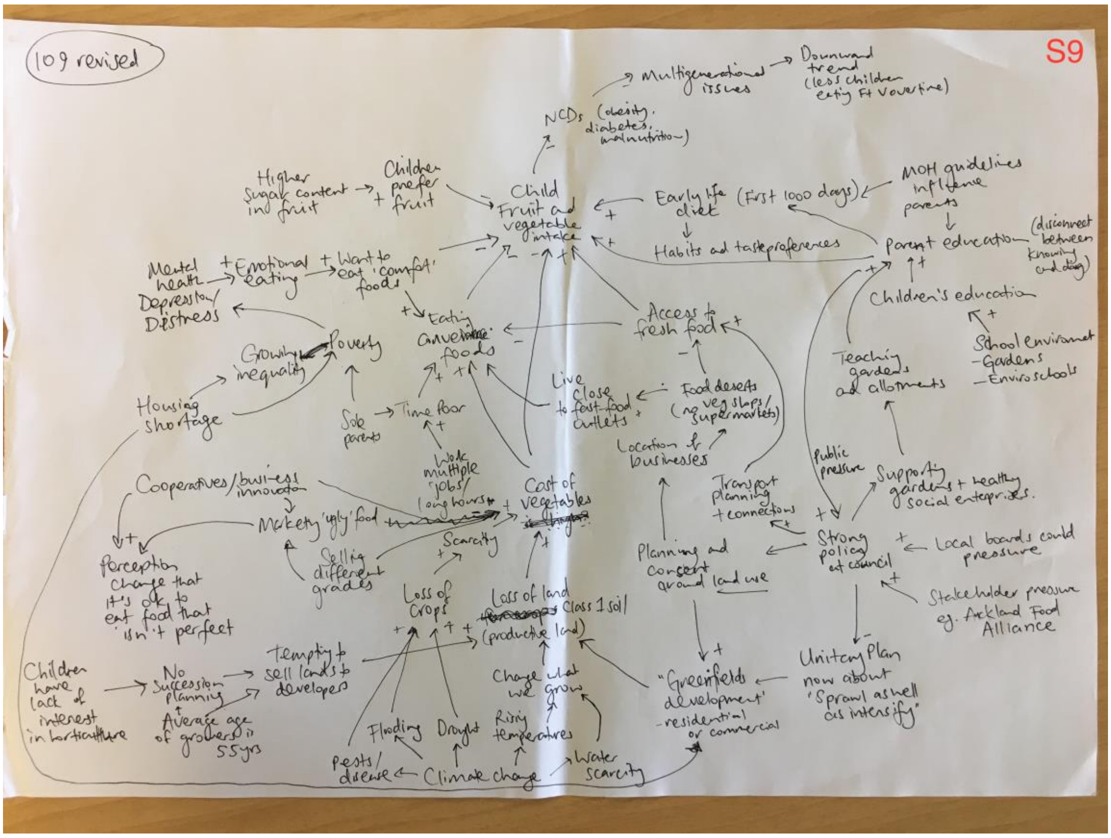


Figure S9. Cognitive map from Interview 109.

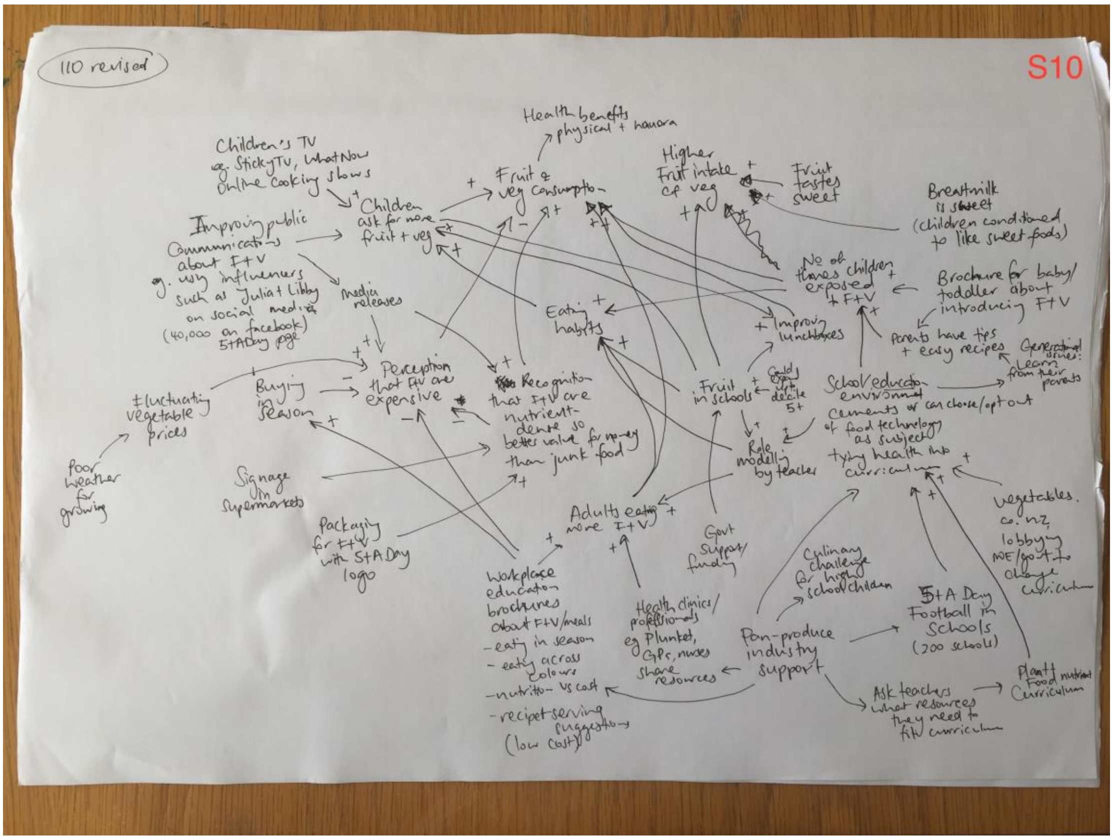


Figure S10: Cognitive map from Interview 110.

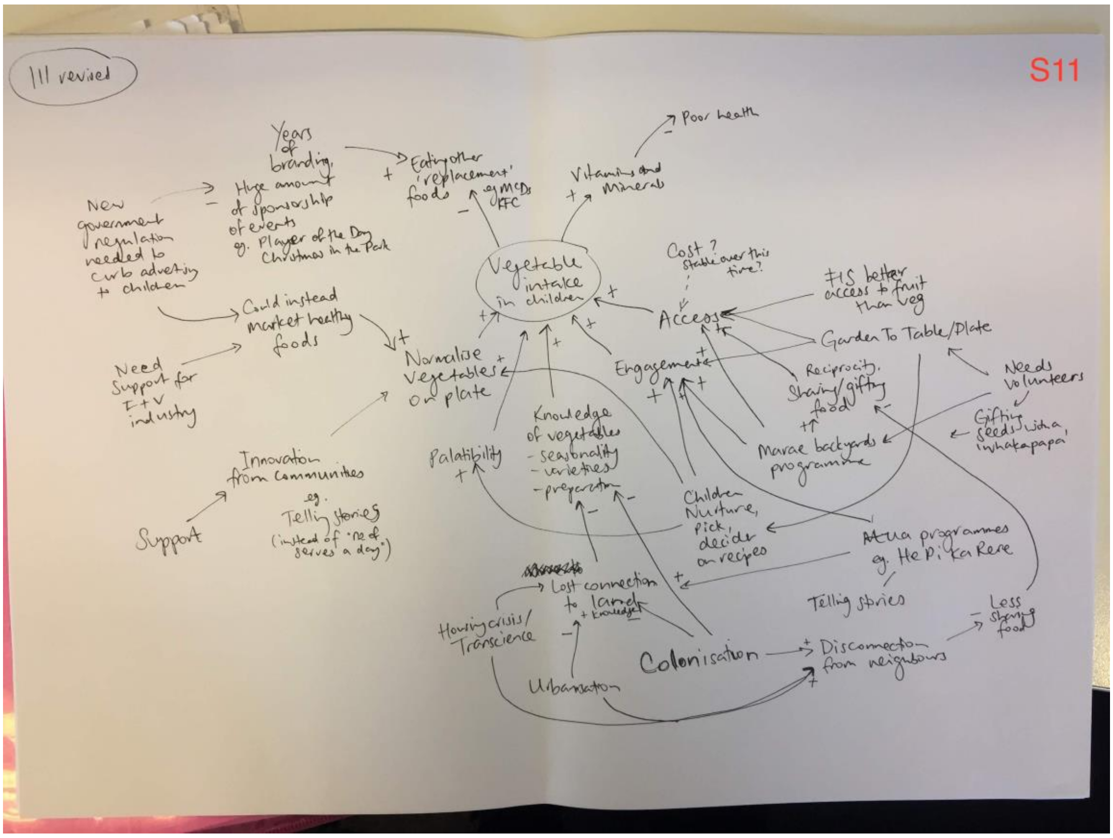


Figure S11. Cognitive map from Interview 111.

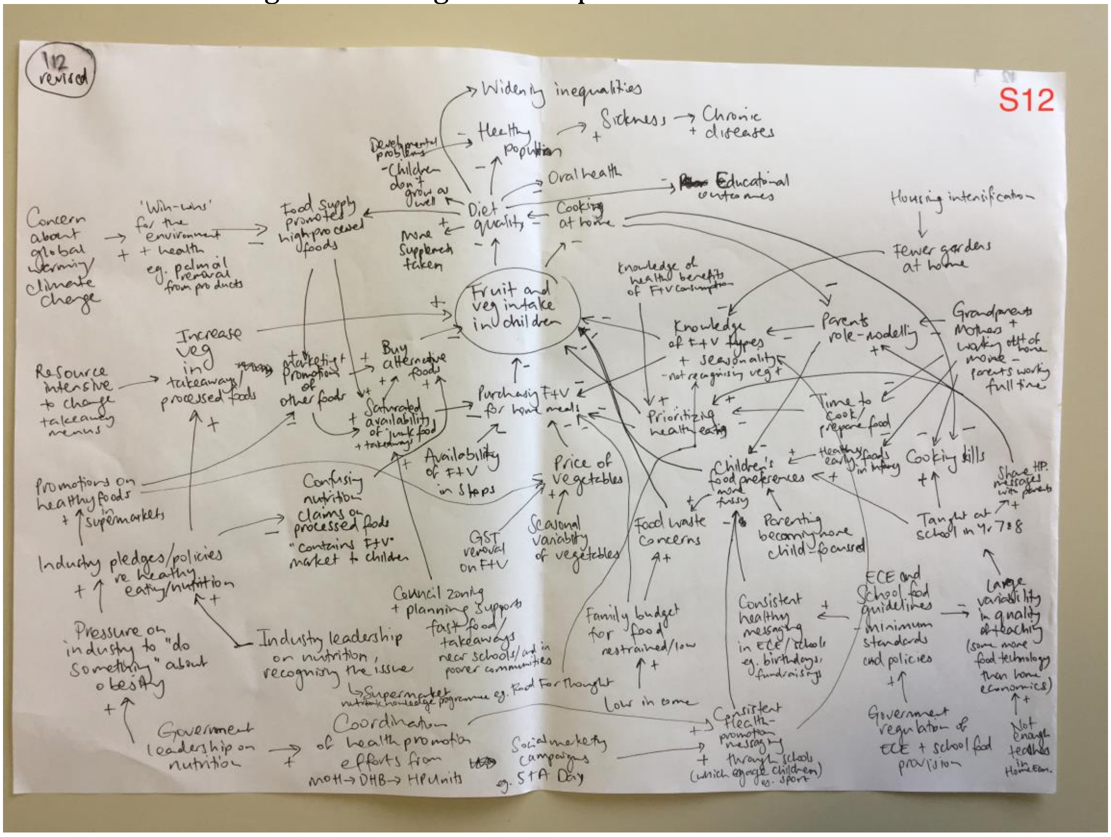


Figure S12: Cognitive map from Interview 112.

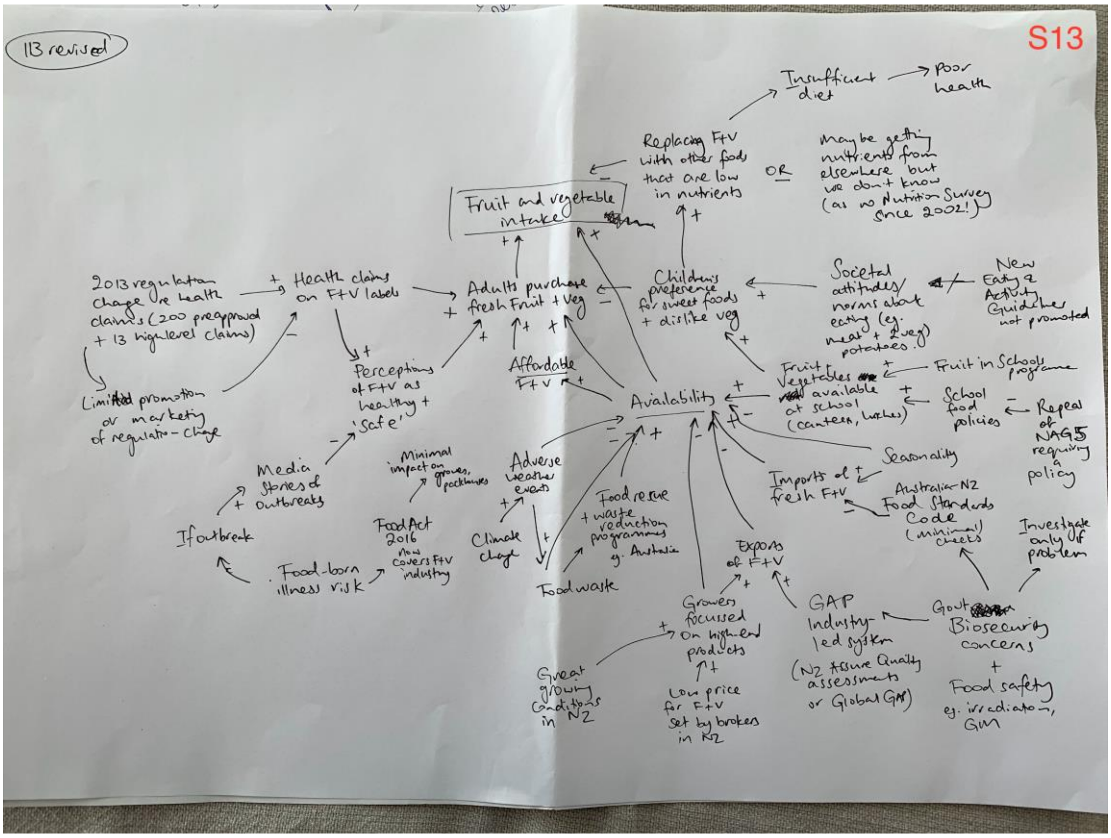


Figure S13. Cognitive map from Interview 113.

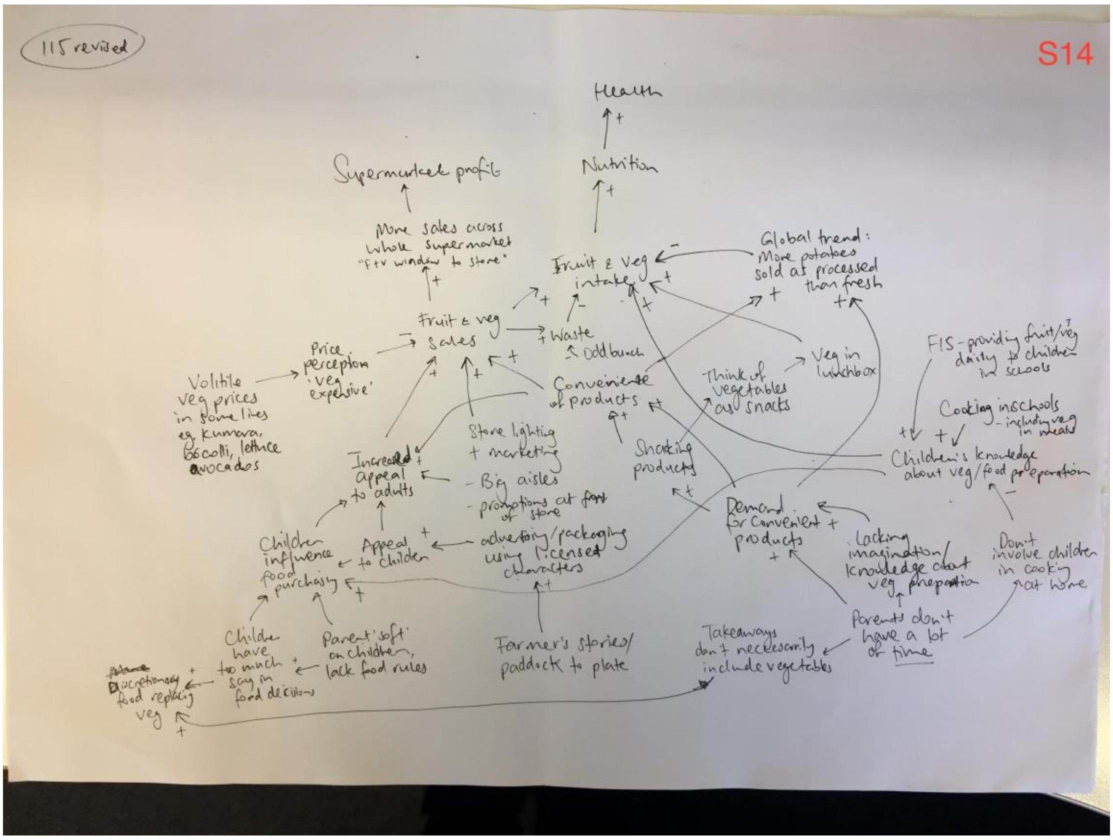


Figure S14. Cognitive map from Interview 115.

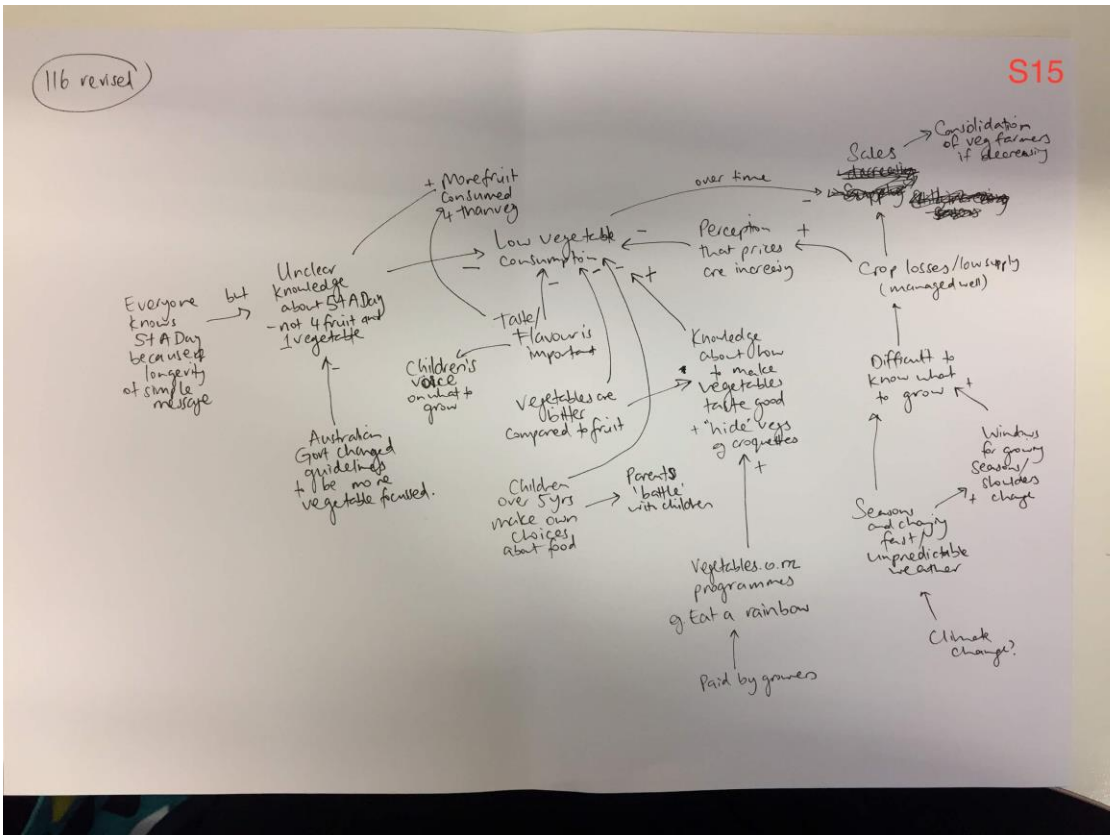


Figure S15. Cognitive map from Interview 116.

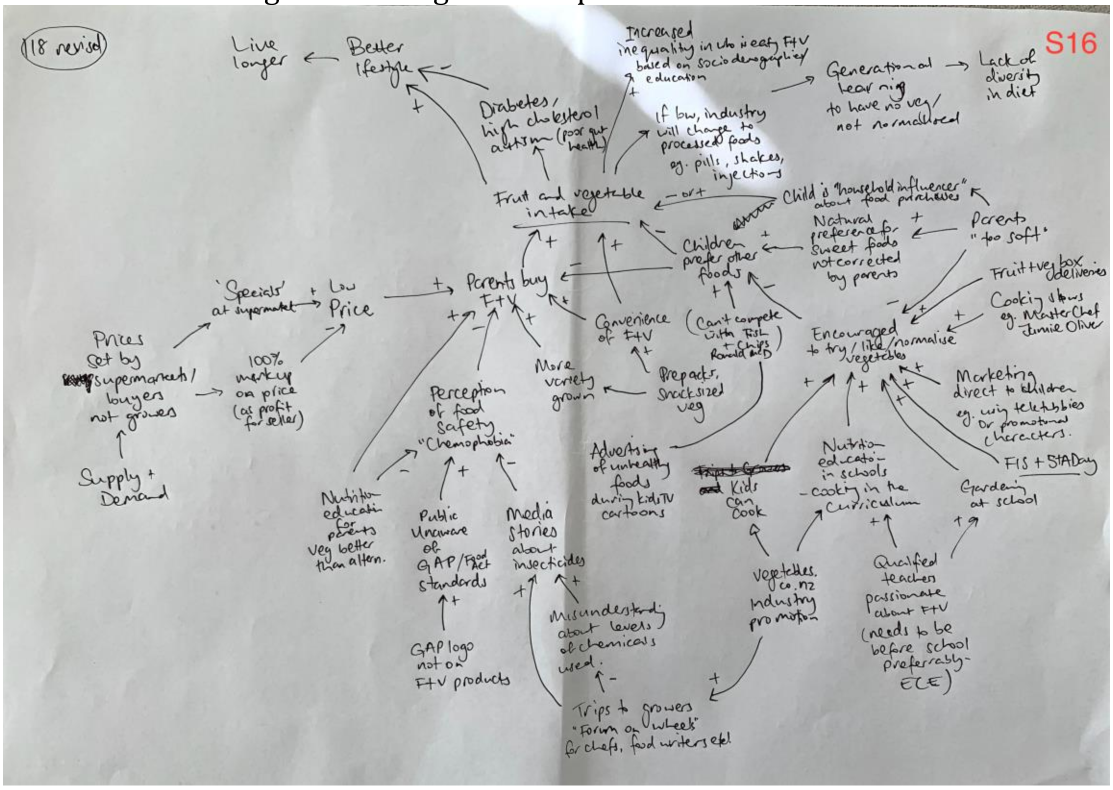


Figure S16. Cognitive map from Interview 118.
